# Infantile Constipation

**Published:** 1887-11

**Authors:** 


### *INFANTILE CONSTIPATION.*

One of the commonest complaints of childhood for which the physician is sought, is constipation. We find it at every stage of infantile life, and sometimes it is exceedingly obstinate. Day says, as causes, there may be:

1. Imperfect digestion and deficiency of glandular secretion from the mucous membrane of the intestinal tract.

2. Deficiency of nerve power, or want of peristalsis, more particularly in the large bowel. This condition is not unfrequently produced by the indiscriminate use of aperient remedies, or may follow a sharp attack of diarrhœa.

3. Dilatation of the intestine from debility, or from flatulent distention, or from fæcal accumulation.

4. Improper diet; as too little liquid food, or too much solid and indigestible material. Many of the patent foods for children, so largely used in the present day, fall under this category. Here may also be mentioned injudicious drugging with iron powders, carbonate of magnesia and the like.

5. Deficient secretion of bile (cold by checking the action of the liver) is a cause of constipation.

6. Malformations of the lower bowel, and painful conditions of the anus, as ulceration, fissure or eczema induced by ascarides, may lead the child to avoid the act of defæcation and to produce habitual constipation.

In a word, most cases of constipation are due to a sluggish state of the mnscular coat of the intestines, to a diminution of the secretion from the mucous membrane or liver, or to improper dietary.

These causes are largely common to adults also, while there are other causes not here enumerated.

A habit of constipation on the part of the nursing mother, not properly attended to, will induce constipation in the child, as will also the habit of purging by active physics. The administration of the pernicious patent nostrums, soothing syrups, cordials, etc., to the fretful child is a potent and serious cause of constipation, as by the influence of the opiates, which they all contain, upon the nervous system of the child, through which the constipation often comes, there is a permanent irritation established, which is sometimes life-long, and often continuing the constipation in spite of all agents not directed to the relief of the irritation.

That the condition of the nervous system is potent as a cause, is proven by the fact that after some prostrating fevers, where there has been great exhaustion of the nervous system, masses of feces have lodged in various parts of the intestinal canal.

This is especially true in chorea, and we know of one child whose mother boasted that she had fed it twenty bottles of Godfrey's cordial, and that no harm came of it, and yet the child suffered from a most intractable form of chorea for several years, and had not entirely recovered at the age of twenty-five years.

Non-attention of the parents of older children to the condition of the child's bowels, is a potent cause of constipation, many parents never inquiring whether or not the child ever evacuates the bowels.

The convulsions of childhood are often due to inpacted masses of fecal matter in the bowels. It may be well to here state also some of the essentials of prevention and cure.

Reasoning from cause to effect, there would be attention to the digestion; to the ingestion of such diet as would be non-irritant and easily digested. If there is deficiency of perastalsis, mild massage of the bowels is often of most marked benefit. We have often advised rubbing the bowels slowly but continuously, after having lubricated the hand, from right to left across the gastric region, down the left side across the lower abdomen to the right side, and up the right side around and around mildly but firmly. This method each morning for several mornings has often overcome a persistent form of the trouble.

All bad habits of the mother, if the child is nursing, must be corrected. It is the radical opinion of the writer that there is *no need of opium in any form* in the child. This may be making a bold assertion, but after several years having elapsed since he has prescribed a dose of the agent in any form, and only good results obtained, he feels justified in the conclusion. Opium is only productive of bad results in the child, and constipation is one of the least of these. Patent soothing remedies should be vehemently denounced; they are pernicious in the extreme.

The parent should cultivate a correct habit on the part of the child, insisting upon regular attention to the bowels at stated times; this will often obviate the difficulty.

But very few medicines should be given. We should study not what to give, but what not to give.

Cascara sagrada, in minute doses, may be given in cases where medicine is absolutely demanded, but it must be given with caution, and discontinued at the earliest moment; at the same time, none of the other measures advised should be neglected.—*Chicago Medical Times.*

